# Gastric cancer radiation therapy: a bibliometric analysis of the scientific literature

**DOI:** 10.3389/fonc.2025.1513255

**Published:** 2025-05-16

**Authors:** Zhen-Hong Weng, Bin-Bin Chen, Jia-Rui Lin, Mu-Ming Xu, Jin-Peng Yuan, Hao-Kai Hu

**Affiliations:** Department of Gastrointestinal Surgery, Cancer Hospital of Shantou University, Medical College, Shantou, Guangdong, China

**Keywords:** gastric cancer, radiotherapy, bibliometrics, R studio, WoSCC

## Abstract

**Background and objectives:**

Gastric cancer is a common malignant tumor primarily treated through surgery. Concurrently, radiation therapy has gained attention as an important local treatment modality. However, its application in gastric cancer remains limited, with ongoing debates on radiation standards. Given that bibliometrics serves as a potent tool to unveil scientific literature, we conducted a bibliometric analysis of literature on radiation therapy for gastric cancer. We explored emerging trends, common patterns in research, tracked collaborations and networks, and anticipated future directions in this clinical context.

**Materials and methods:**

We searched the electronic Web of Science (WOS) database using keywords “gastric cancer” and “radiation therapy” for manuscripts published in English from 2014 to 2023. Data analysis was conducted using R-Studio software, employing bibliometric methods based on the bib liometrix R package. Quantification involved assessing the most relevant authors based on document production and citation metrics. Author productivity was analyzed using Lotka’s law. Main thematic areas included isolated (niche) topics, emerging topics, hot (motor) topics, and necessary (basic) topics.

**Results:**

A total of 2405 documents were initially retrieved, from which 484 articles closely related to gastric cancer radiation therapy were selected, showing an annual growth rate of -2.05%. Overall, publications were found in 186 different journals, with “FRONTIERS IN ONCOLOGY” being the most relevant journal. The most prolific authors were from South Korea. Clinical trials (survival, phase III clinical trials) and treatment strategies (surgery, chemotherapy, radiation therapy, perioperative treatment) represented the fundamental topics. Emerging topics included radiation dose, therapeutic response and immunotherapy.

**Conclusion:**

Radiation therapy for gastric cancer has evolved in terms of timing, modes, target sites, and emerging combination therapies. It benefits patients with potentially resectable, unresectable, or isolated distant metastases. Immunotherapy combined with radiation shows significant potential and could become a new breakthrough in treatment strategies.

## 1 Introduction

Gastric cancer (GC) is one of the most significant contributors to the global cancer burden. According to the “Global Cancer Data Report 2022,” gastric cancer had a high number of new cases in 2022, ranking as the fifth most common cancer (4.9%) after lung cancer (2.48 million, 12.4%), female breast cancer (2.31 million, 11.6%), colorectal cancer (1.93 million, 9.6%), and prostate cancer (1.47 million, 7.3%). Due to the often late-stage diagnosis of gastric cancer, the mortality rate is alarmingly high. It is estimated that approximately 660,000 people worldwide died from gastric cancer in 2022, making it the fourth leading cause of cancer-related deaths globally (1).

Currently, radical surgery is still considered the only potentially curative treatment for gastric cancer. Over the years, survival rates and incidence have improved due to the adoption of radical surgical approaches. However, after surgical resection for gastric cancer, both local and distant recurrence rates remain high (2).The high recurrence rate makes gastric cancer a challenging disease to cure solely through surgery. Moreover, lacking typical clinical precursors for gastric cancer, over 75% of patients are diagnosed at an advanced stage. The survival rate for these patients with locally advanced gastric cancer ranges only from 20% to 50%. Approximately 50% of patients with locally advanced gastric cancer lose their opportunity for surgical treatment. Therefore, researchers have been exploring adjuvant therapies for gastric cancer following surgical resection, such as radiation therapy (RT), chemotherapy, and chemoradiotherapy. Consequently, comprehensive treatment focusing on radiation therapy (RT) and chemotherapy has gained significant attention in recent years.

Numerous scientists have dedicated their efforts to postoperative radiotherapy research, with the most famous being the Intergroup 0116 (INT-0116) study (3), renowned for its well-designed and large sample content. It has been demonstrated that postoperative chemoradiotherapy (fluorouracil and leucovorin followed by radiation therapy) improves the 5-year overall survival rate and reduces the rate of local recurrence compared to surgery alone. Since then, an increasing number of studies have sought to confirm the survival benefits of adjuvant chemoradiotherapy. However, the results of these trials have either been contradictory or inconclusive due to their relatively limited patient recruitment and varying inclusion criteria. As a result, the role of radiation therapy in the adjuvant treatment of gastric cancer following surgery remains controversial.

Preoperative radiotherapy is primarily used to reduce the tumor burden in patients with advanced gastric cancer. This process can render previously inoperable patients eligible for surgery. Additionally, preoperative radiotherapy may play a unique role in controlling micrometastases, and the pathological response observed post-radiotherapy can provide significant prognostic information. Several major clinical trials have demonstrated that cancer of the gastroesophageal junction (GEJ) has yielded better therapeutic outcomes from preoperative radiotherapy compared to gastric cancer. Stahl et al. found that preoperative radiotherapy significantly increased the pathological complete response rate for GEJ adenocarcinoma (15.6% vs. 2.0%) and improved the 3-year overall survival (OS) rate (47.4% vs. 27.7%, P=0.07) (4). Similarly, Hagen and colleagues investigated 366 cases of gastric or GEJ cancer and found that patients receiving preoperative chemoradiotherapy (carboplatin + paclitaxel, 5 weeks; 41.4 Gy/23 fractions, 5 days/week) had a significantly higher resection rate (92% vs. 69%, P < 0.001) and OS (49.4 months vs. 24 months, median survival) compared to those receiving surgery alone (5). Furthermore, preoperative chemoradiotherapy was associated with reduced local recurrence rates (LRR, 14% vs. 34%, P < 0.001) and distant metastasis rates (29% vs. 35%, P = 0.025) when compared to surgery alone. This regimen has become the recommended treatment for GEJ adenocarcinoma in the United States.

Common local symptoms in patients with gastric cancer include obstruction, bleeding, or pain. Interventions to alleviate these symptoms encompass palliative radiotherapy (RT), palliative chemotherapy, gastric bypass surgery, palliative gastrectomy, and endoscopic stent placement. Radiotherapy serves as a non-invasive treatment option for these local symptoms. Literature suggests that two-thirds of patients receiving radiotherapy will experience clinical benefits, with the highest response rate observed in hemorrhage control (6).Low Biologically Effective Dose (BED) regimens appear sufficient to alleviate symptoms. However, the optimal dose-fractionation scheme for symptom relief remains unclear.

Given that bibliometrics is a powerful tool for uncovering scientific literature on specific topics over defined time spans, we decided to conduct a bibliometric analysis of the radiotherapy literature for gastric cancer published over the past decade. Through this current analysis, we aim to explore emerging trends and common patterns in research, track collaboration and networks, and predict future directions for clinical research in radiation oncology as applied to gastric cancer.

## 2 Materials and methods

### 2.1 Literature search strategy

Web of Science (WoS) is recognized as one of the most comprehensive, systematic, and authoritative databases, encompassing a vast array of literature metrics and over 12,000 high-quality journals from around the world. It is widely utilized for bibliometric analysis and visualization of scientific literature.

**Figure 1:**
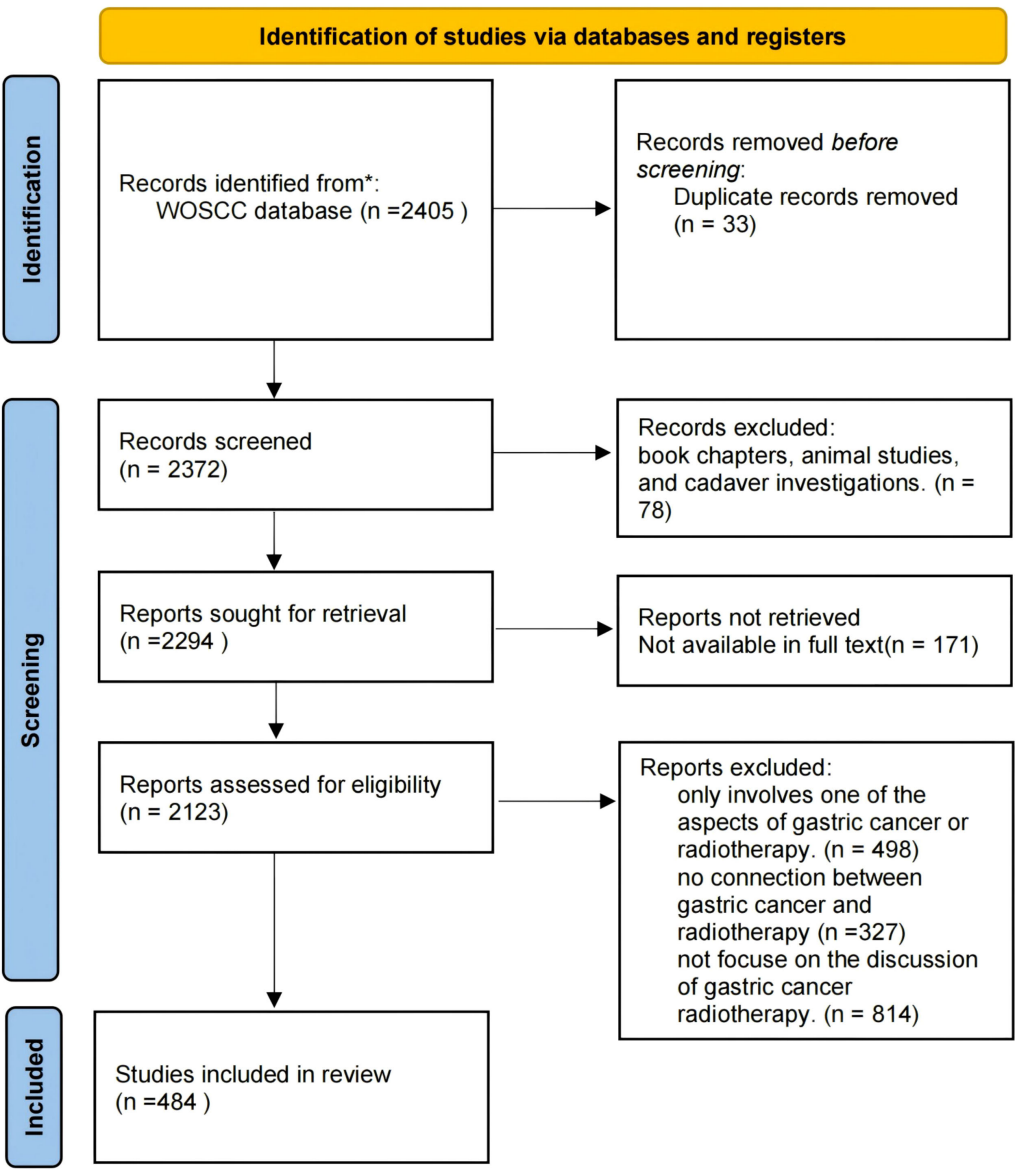
PRISMA flowchart for literature screening.

The literature screening process included in this study follows the PRISMA guidelines, as depicted in **Figure 1**. Publications were retrieved from the Science Citation Index Expanded (SCI-Expanded) of WoSCC for the period spanning from January 1, 2014 to October 25, 2023. Data were downloaded on October 25, 2023, within a single day to mitigate biases arising from daily database updates. Search terms used were: ((((((((((((((((((((ALL=(Neoplasm, Stomach)) OR ALL=(Stomach Neoplasm)) OR ALL=(Neoplasms, Stomach)) OR ALL=(Gastric Neoplasms)) OR ALL=(Gastric Neoplasm)) OR ALL=(Neoplasm, Gastric)) OR ALL=(Neoplasms, Gastric)) OR ALL=(Cancer of Stomach)) OR ALL=(Stomach Cancers)) OR ALL=(Gastric Cancer)) OR ALL=(Cancer, Gastric)) OR ALL=(Cancers, Gastric)) OR ALL=(Gastric Cancers)) OR ALL=(Stomach Cancer)) OR ALL=(Cancer, Stomach)) OR ALL=(Cancers, Stomach)) OR ALL=(Cancer of the Stomach)) OR ALL=(Gastric Cancer, Familial Diffuse) AND (((((((((((((((((TS=(Radiotherapy)) OR ALL=(Radiotherapies)) OR ALL=(Radiation Therapy)) OR ALL=(Radiation Therapies)) OR ALL=(Therapies, Radiation)) OR ALL=(Radiation Treatment)) OR ALL=(Radiation Treatments)) OR ALL=(Treatment, Radiation)) OR ALL=(Radiotherapy, Targeted)) OR ALL=(Radiotherapies, Targeted)) OR ALL=(Targeted Radiotherapies)) OR ALL=(Targeted Radiotherapy)) OR ALL=(Targeted Radiation Therapy)) OR ALL=(Radiation Therapies, Targeted)) OR ALL=(Targeted Radiation Therapies)) OR ALL=(Therapies, Targeted Radiation)) OR ALL=(Therapy, Targeted Radiation)) OR ALL=(Radiation Therapy, Targeted).

### 2.2 Inclusion and exclusion criteria

Inclusion criteria encompassed: (1)English-language articles, reviews and meta-analyses pertaining to radiotherapy for gastric cancer; and (2) only published data were used.

Exclusion criteria included conference abstract, editorial material, book chapters, animal studies, and cadaver investigations.

### 2.3 Data extraction and visualization methods

Data collection: All relevant details were retrieved from the Web of Science in text format, encompassing author names, article categories, citation counts, countries, digital object identifiers (DOIs), impact factors, journal names, institutions, keywords, sample sizes, study designs, titles, and publication years.

Data analysis: Data were analyzed using R-studio for summarizing and visualizing scientific literature. R-studio provides a rich set of literature analysis packages and user-friendly syntax for efficiently processing large-scale literature data, extracting valuable insights, and uncovering developmental trends and key influencing factors in academic fields. The main steps are as follows: (1) importing the dataset into R software, (2) selecting the type of analysis (i.e., “co-occurrence network,” “thematic evolution,” and “main information”), and (3) setting analysis parameters.

## 3 Results

### 3.1 Overview

**Figure 2:**
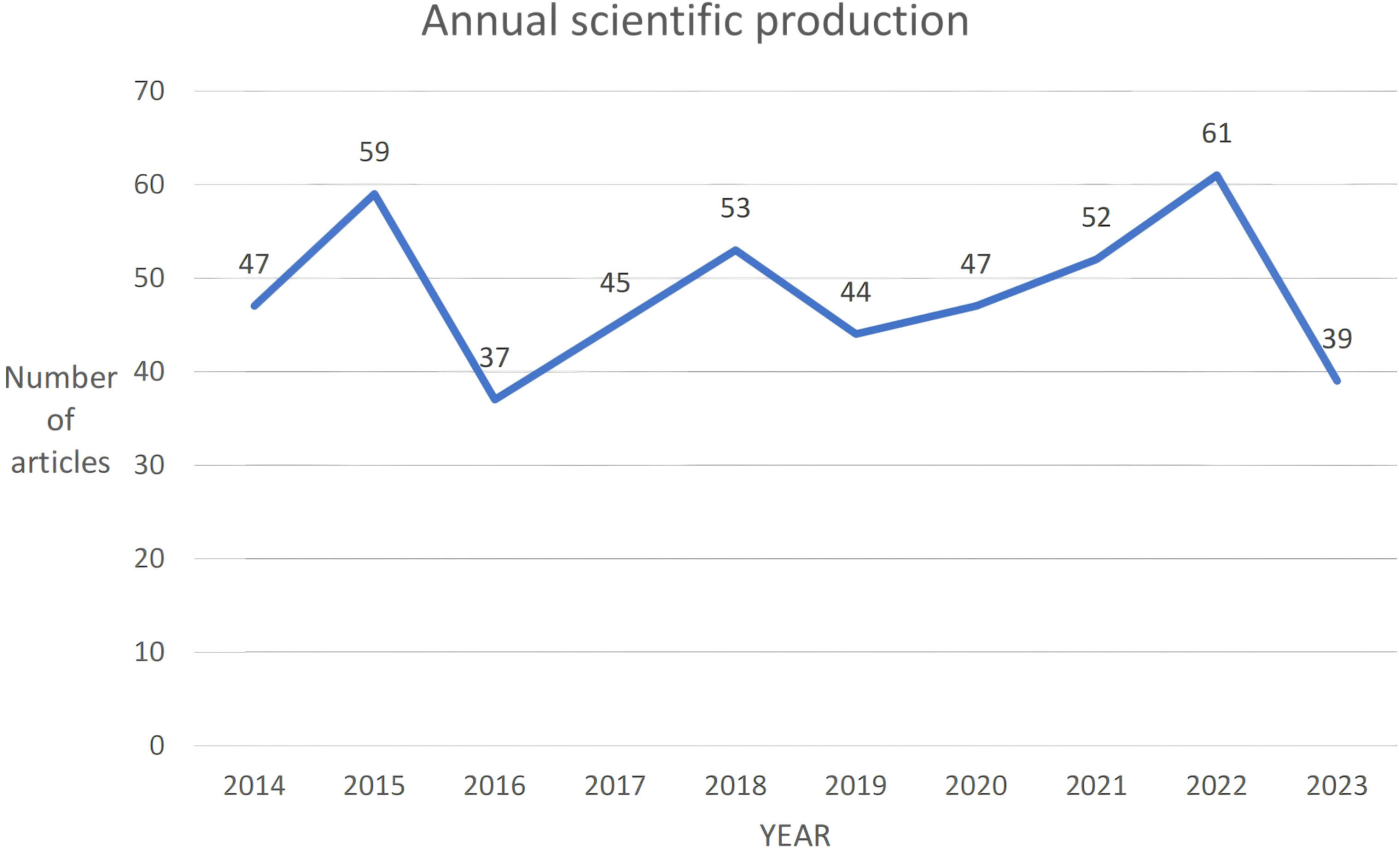
Annual scientific production.

From 2014 to 2023, a total of 484 articles were collected. Overall, these articles involved 2940 authors, averaging 8 authors per publication. There was a negative annual growth rate of -2.05%, resulting in a decrease in scientific output from 47 articles in 2014 to 39 articles in 2023 (**Figure 2**). The average annual citations per article remained stable between 2 and 3 citations, with peaks in 2015 (2.46 citations per article) and 2019 (2.98 citations per article). The highest average citation per article was observed in 2015 at 24.63 citations, while the lowest was in 2023 at 0.54 citations, indicating a shorter duration of citable relevance.

### 3.2 Journals

**Figure 3:**
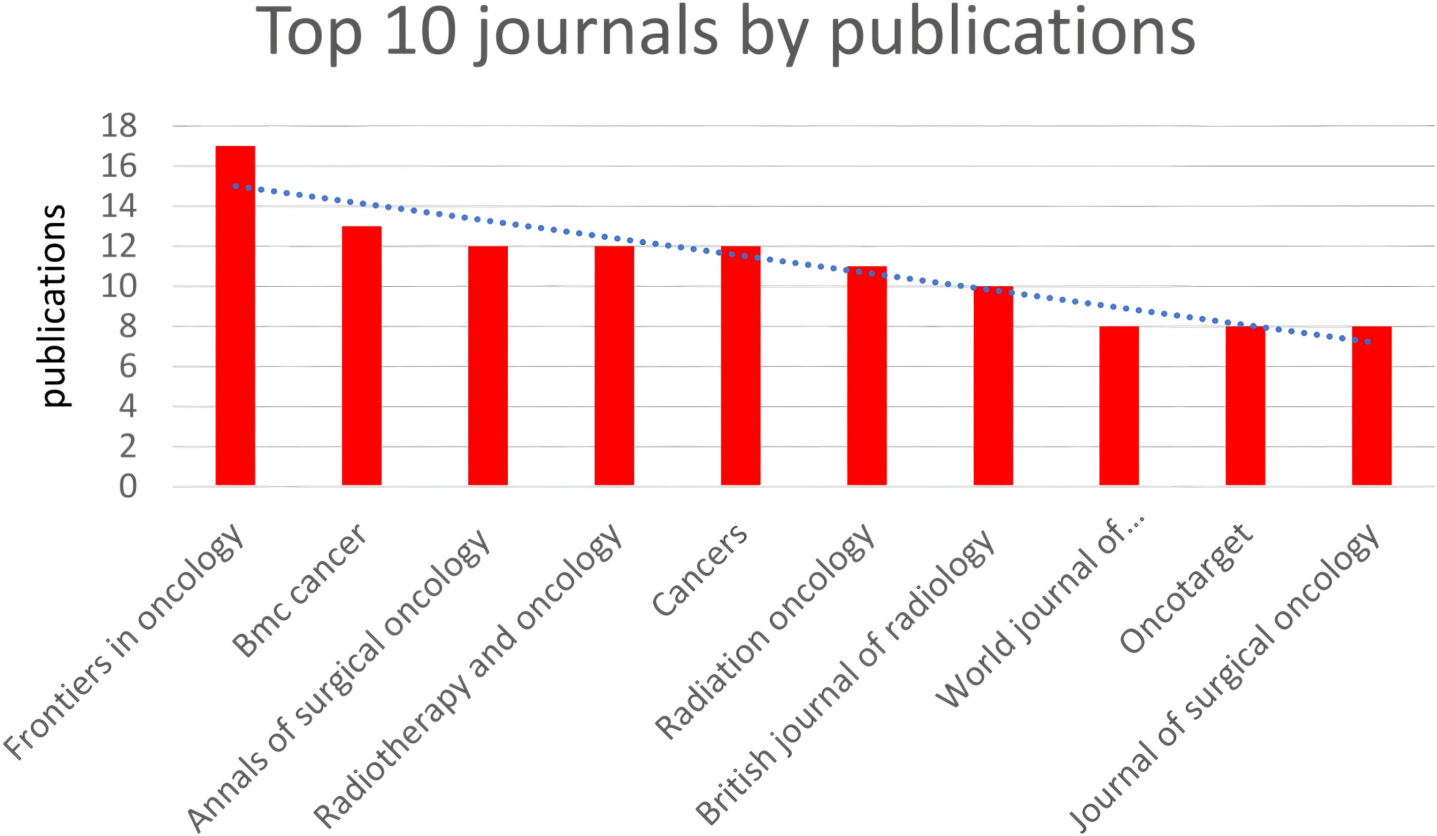
Top 10 journals by publications.

In total, 186 journals published one or more of these articles. **Table 1** and **Figure 3** summarize the top 10 journals by publication volume and their basic information. Impact Factor (IF), often used to measure a journal’s importance within its field, represents the 5-year average number of citations. 17 journals (core sources) published approximately one-third of the retrieved documents. The most relevant journal was “FRONTIERS IN ONCOLOGY,” which published 17 articles from 2014 to 2023, followed by “BMC CANCER” (n = 13), “ANNALS OF SURGICAL ONCOLOGY” (n = 12), “CANCERS” (n = 12), and “RADIOTHERAPY AND ONCOLOGY” (n = 12). These journals predominantly fall within Q2, with impact factors around 5. Similar to most research fields, articles in the field of gastric cancer radiotherapy are mostly exploratory, with few achieving significant breakthroughs or providing high-level evidence in evidence-based medicine. Overall, the quality of articles in the field of gastric cancer radiotherapy remains good, with most articles located in the Q2 JCR zone.

**Table 1:** Summarizes the top 10 journals with the highest publication.

Rank	Journal	Country	Publication	Total Citations	Total link strength	CR	IF (5years)
1	Frontiers in oncology	SWITZERLAND	17	43	23	Q2	5.2
2	Bmc cancer	ENGLAND	13	244	16	Q2	4.3
3	Annals of surgical oncology	USA	12	189	22	Q2	4.4
4	Radiotherapy and oncology	NETHERLANDS	12	178	28	Q1	5.8
5	Cancers	SWITZERLAND	12	41	17	Q2	5.6
6	Radiation oncology	ENGLAND	11	100	13	Q2	3.8
7	British journal of radiology	ENGLAND	10	52	17	Q3	3.1
8	World journal of gastroenterology	USA	8	605	13	Q2	5.3
9	Oncotarget	USA	8	82	13	Q2	5.312
10	Journal of surgical oncology	USA	8	61	15	Q4	3.1

**Figure 4:**
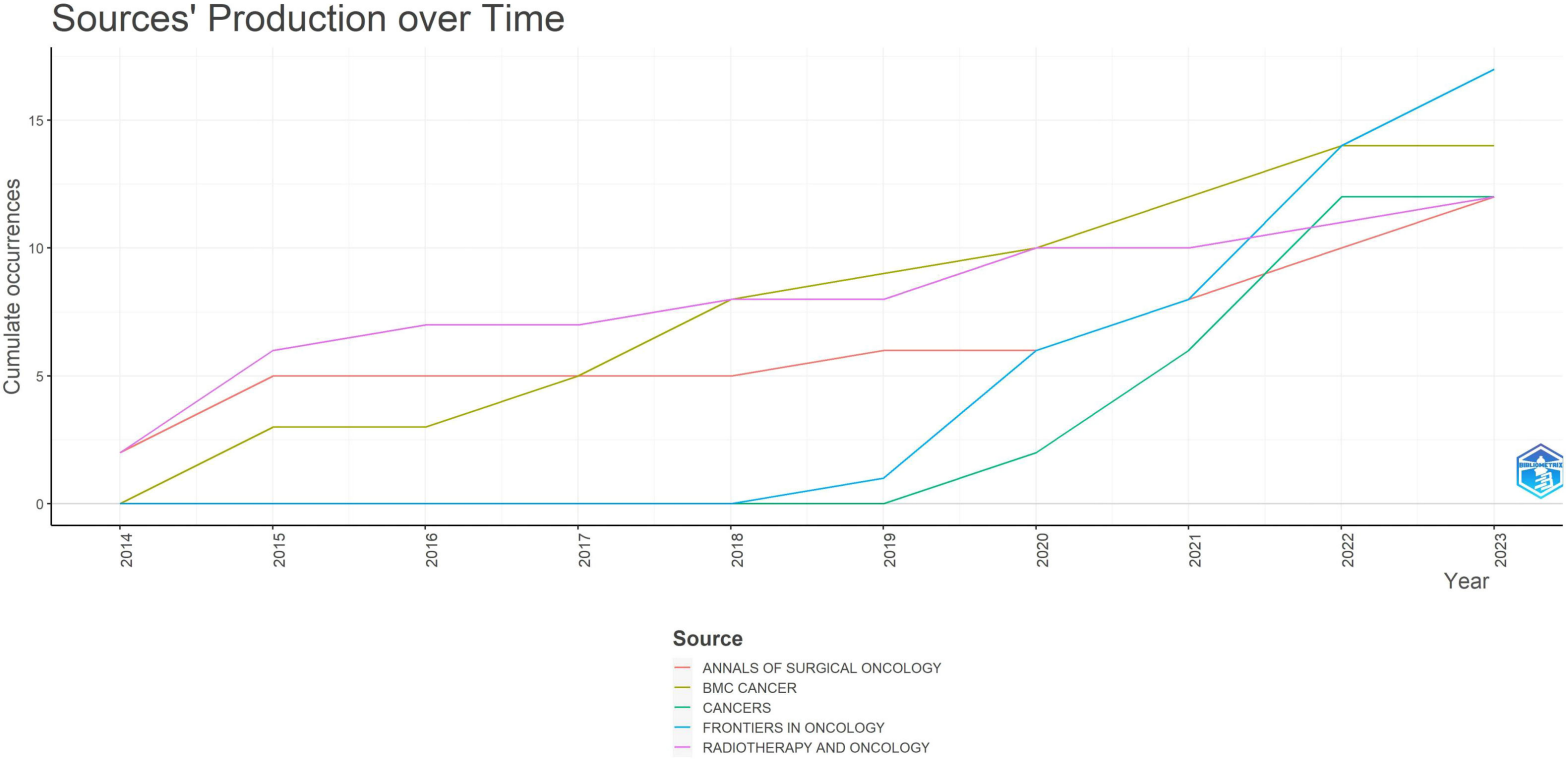
Sources production over time.

**Figure 5:**
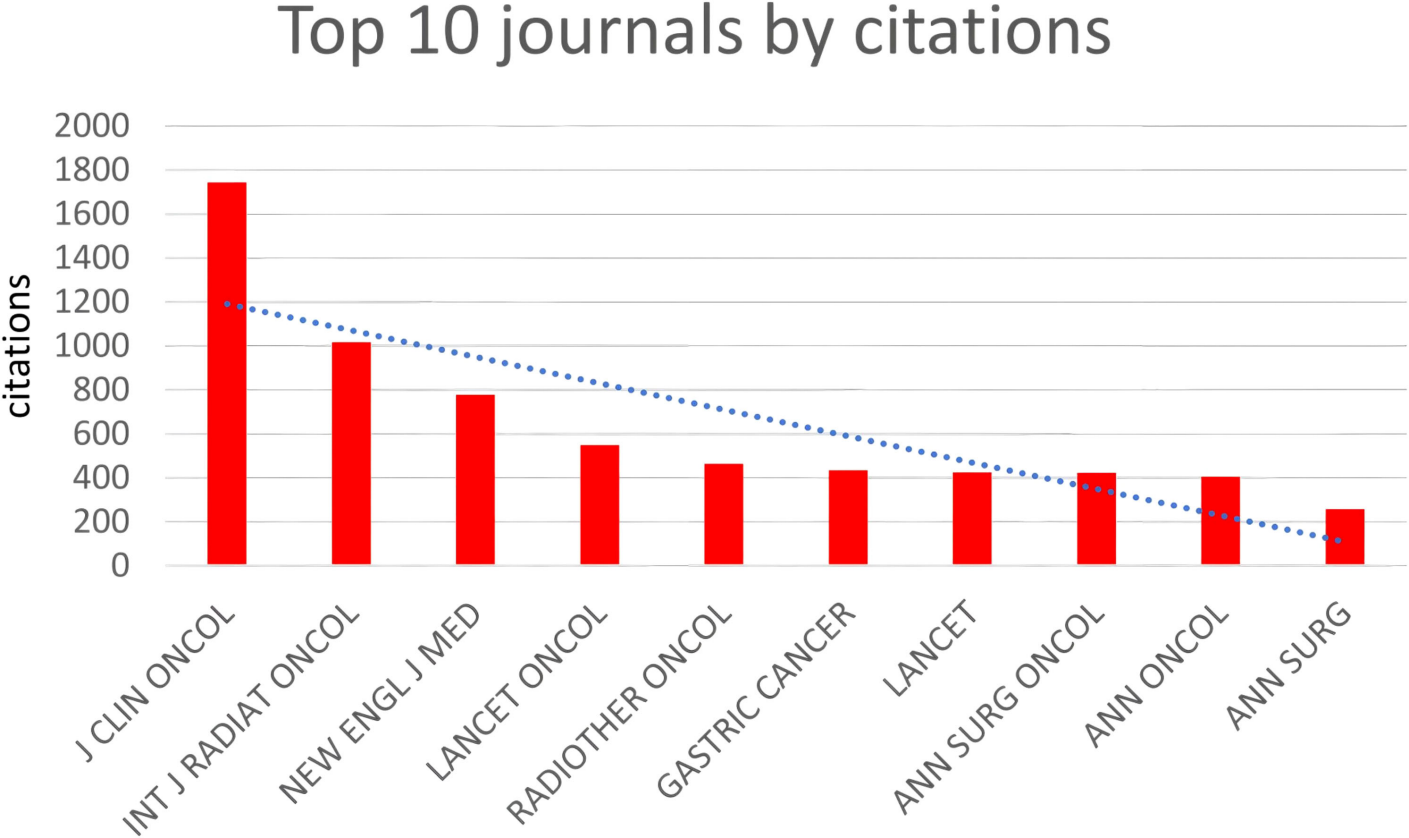
Top 10 journals by citations.

**Figure 4** illustrates the dynamics of the top 5 relevant journals. Among the top 5 journals, “FRONTIERS IN ONCOLOGY” and “CANCER” saw a rapid increase in published articles starting from 2018 and 2019 respectively, both originating from Switzerland. In contrast, the publication rate of the other three journals remained relatively stable. This highlights how “FRONTIERS IN ONCOLOGY” and “CANCER” have gathered excellent articles discussing gastric cancer radiotherapy in recent years.

(**Table 2**, **Figure 5**) The most cited journals among all 484 articles (from the reference list) were “J CLIN ONCOL” with 1744 citations, followed by “INT J RADIAT ONCOL” with 1017 citations, “NEW ENGL J MED” with 779 citations, “LANCET ONCOL” with 548 citations, and “RADIOTHER ONCOL” with 464 citations. These journals are globally recognized top-tier publications that collect the highest quality articles on gastric cancer radiotherapy to date, many of which are considered landmark articles worthy of citation and reference by researchers worldwide. “J CLIN ONCOL” focuses on clinical oncology, while “INT J RADIAT ONCOL” specializes in radiation oncology, ranking first and second respectively among cited journals, underscoring their profound influence on the application of radiotherapy in gastric cancer.

**Table 2:** Summarizes the top 10 journals with the highest citations.

Rank	Journal	Citations	IF
1	J CLIN ONCOL	1744	37.7
2	INT J RADIAT ONCOL	1017	6.4
3	NEW ENGL J MED	779	115.7
4	LANCET ONCOL	548	46.1
5	RADIOTHER ONCOL	464	5.8
6	GASTRIC CANCER	434	7.5
7	LANCET	424	118.1
8	ANN SURG ONCOL	423	4.4
9	ANN ONCOL	405	32.4
10	ANN SURG	258	10.8

### 3.3 Authors, affiliations, countries

The most prolific authors in terms of publications are Lee Jeeyun, Kang Won Ki, and Lim Do Hoon, with 11, 10, and 10 articles respectively. Among the top 10 authors by publication volume, they hail from South Korea, the Netherlands, and China. South Korea is the most productive in this field, primarily represented by Samsung Medical Center (**Table 3**).

**Table 3:** Summarizes the top 10 authors with the highest volume of publications.

Rank	Author	Publication	Total Citations	Country	Affiliation
1	lee, jeeyun	11	367	KOREA	Samsung medical center
2	kang,won ki	10	367	KOREA	Samsung medical center
3	lim, do hoon	10	361	KOREA	Samsung medical center
4	cats, annomieke	9	476	NETHERLAND	Netherland cancer institute
5	verhei, marcel	9	476	NETHERLAND	University of amsterdam
6	kim, seung tae	9	362	KOREA	Samsung medical center
7	park, se hoon	9	358	KOREA	Samsung medical center
8	sohn, tae sung	9	358	KOREA	Samsung medical center
9	choi, min gew	9	358	KOREA	Samsung medical center
10	wang,xin	9	33	CHINA	Peking union medical college

**Figure 6:**
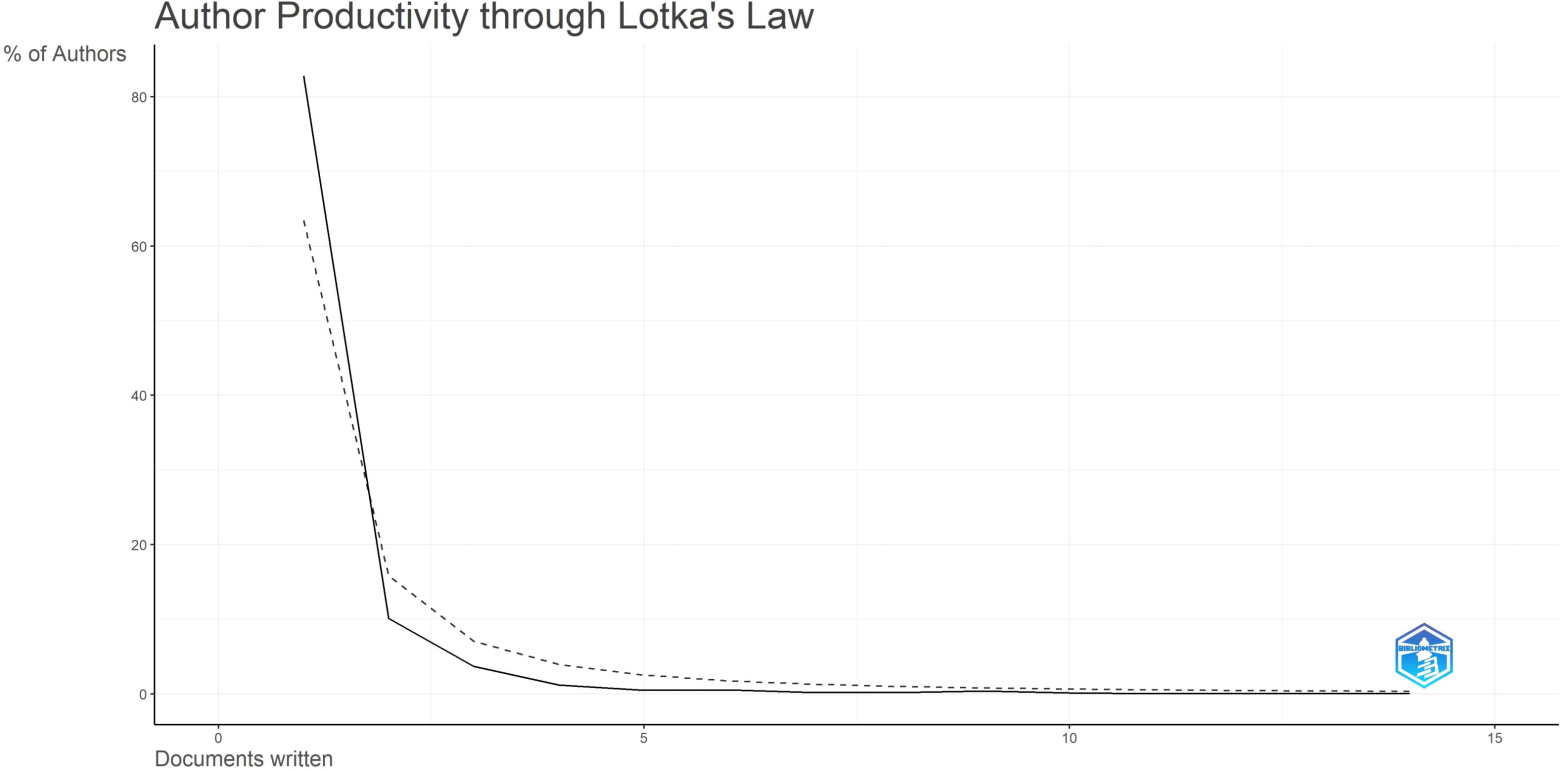
Author productivity through Lotka’s law.

According to Lotka’s Law (7, 8)(**Figure 6**), the frequency distribution of scientific productivity identified 114 “core” authors who contributed to at least 3 articles (2.48%) and 2435 “occasional” authors who published only one paper (82.8%). **Table 4** calculates the number of articles published by country based on the corresponding authors, with China topping the list. SCP (Single Country Publications) denotes articles where all authors are from the same country; MCP (Multiple Country Publications) indicates articles with authors from multiple countries, reflecting international collaboration. MCP Ratio = MCP/Articles denotes the proportion of collaborative articles in a country’s total publications. A higher value indicates more frequent international collaboration. According to MCP ratios, Turkey and major East Asian countries (China, Japan, South Korea) show relatively low rates of international collaboration, whereas North America (USA, Canada) and Europe (Netherlands, Germany, Italy) demonstrate higher rates. The national collaboration network based on publications shows the collaboration patterns between countries, with line thickness indicating the degree of collaboration closeness. Close collaborations are observed between the USA and Canada, USA and China, China and Singapore, and among European countries.

**Table 4:** Summarizes the top 10 countries with the highest volume of publications (Calculated by corresponding author).

Rank	Country	Articles	SCP	MCP	Freq	MCP_Ratio
1	CHINA	211	193	18	0.436	0.085
2	USA	72	62	10	0.149	0.139
3	JAPAN	29	27	2	0.06	0.069
4	KOREA	27	25	2	0.056	0.074
5	NETHERLANDS	20	15	5	0.041	0.25
6	TURKEY	19	18	1	0.039	0.053
7	ITALY	13	11	2	0.027	0.154
8	CANADA	11	8	3	0.023	0.273
9	GERMANY	11	4	7	0.023	0.636
10	IRAN	8	8	0	0.017	0

**Figure 7:**
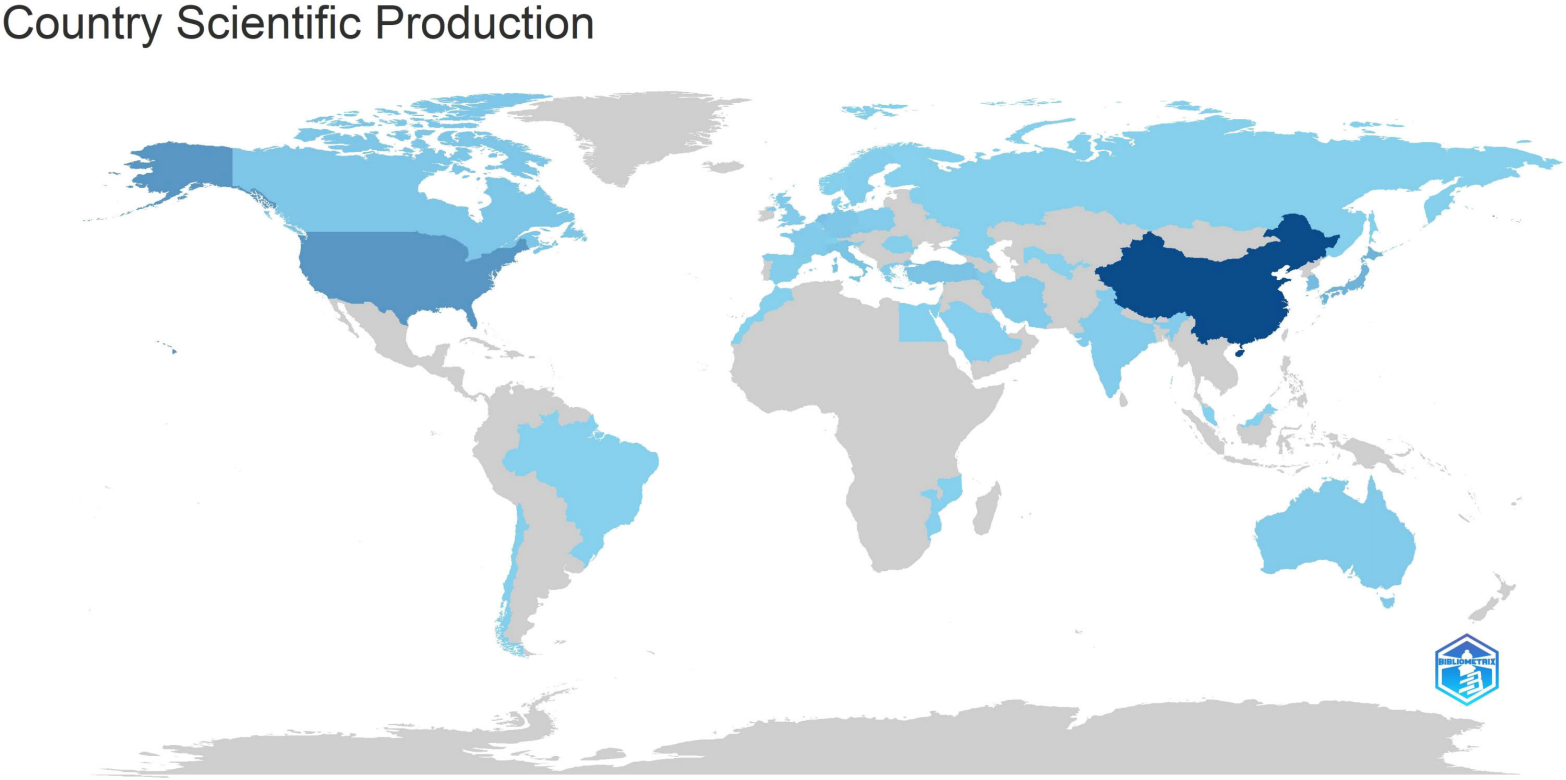
Country scientific production.

**Table 5** and **Figure 7** illustrate the production outputs by specific countries, with China leading with a total of 712 articles. In **Figure 7**, darker colors indicate that the country has a higher publication volume. Significant affiliated institutions include China (Fudan University, Peking Union Medical College, Chinese Academy of Medical Sciences - Peking Union Medical College), South Korea (Samsung Medical Center, Sungkyunkwan University (SKKU)), followed by the USA (University of Texas System, UTMD Anderson Cancer Center, Harvard University), and the Netherlands (Netherlands Cancer Institute) (**Table 6**).

**Table 5:** Summarizes the top 10 countries with the highest volume of publications (Calculated by all authors).

Rank	Country	Articles
1	CHINA	712
2	USA	300
3	JAPAN	145
4	SOUTH KOREA	103
5	NETHERLANDS	83
6	TURKEY	67
7	ITALY	65
8	CANADA	54
9	GERMANY	53
10	CZECH REPUBLIC	39

**Table 6:** Summarizes the top 10 affiliations with the highest volume of publications.

Rank	Affiliation	Articles
1	SAMSUNG MEDICAL CENTER	43
2	FUDAN UNIVERSITY	41
3	PEKING UNION MEDICAL COLLEGE	40
4	CHINESE ACADEMY OF MEDICAL SCIENCES - PEKING UNION MEDICAL COLLEGE	37
5	SUNGKYUNKWAN UNIVERSITY (SKKU)	37
6	UNIVERSITY OF TEXAS SYSTEM	36
7	UNIVERSITY OF TORONTO	35
8	UTMD ANDERSON CANCER CENTER	33
9	HARVARD UNIVERSITY	27
10	NETHERLANDS CANCER INSTITUTE	27

### 3.4 Articles

The **Table 7** lists the top 20 most cited articles in the field of gastric cancer (6, 9–27). Among these twenty papers, 13 are original articles, with 4 being clinical trials; 6 are systematic reviews/commentary articles, and 1 is a clinical practice guideline/consensus. The top-ranked article is “Treatment of gastric cancer,” cited globally 474 times. Most of these articles (n = 16) were published between 2014 and 2018, with limited recent publications (2019-2023), possibly due to a shorter citation window.

**Table 7:** Summarizes the top 20 most cited articles in the field of gastric cancer.

Paper	DOI	Total Citations	TC per Year
ORDITURA M (9), WORLD J GASTROENTERO	10.3748/wjg.v20.i7.1635	474	43.09
CATS A (10), LANCET ONCOL	10.1016/S1470-2045(18)30132-3	323	46.14
PARK SH (11), J CLIN ONCOL	10.1200/JCO.2014.58.3930	290	29.00
WANG FH (12), CANCER COMMUN	10.1002/cac2.12193	205	51.25
MURO K (13), ANN ONCOL	10.1093/annonc/mdy502	141	23.50
LEONG T (14), BMC CANCER	10.1186/s12885-015-1529-x	114	11.40
WALTERS S (15), BRIT J CANCER	10.1038/bjc.2015.265	84	8.40
FUCHS CS (16), J CLIN ONCOL	10.1200/JCO.2017.74.2130	81	10.13
VRÁNA D (2019) (17), INT J MOL SCI	10.3390/ijms20010013	76	12.67
WANG JP (18), ANN SURG ONCOL	10.1245/s10434-015-4388-4	71	7.10
TORII K (19), GASTRIC CANCER	10.1007/s10120-014-0395-6	67	6.70
COHEN DJ (20), J CLIN ONCOL	10.1200/JCO.2014.59.7765	65	6.50
MARTÍNEZ-CARMONA M (21), J MATER CHEM B	10.1039/c5tb00304k	65	6.50
IZUISHI K (22), J GASTROINTEST LIVER	10.15403/jgld.2014.1121.251.rv2	55	6.11
COCCOLINI F (23), WORLD J GASTROENTERO	10.3748/wjg.v22.i3.1139	55	6.11
ZHANG XK (24), ONCOL REP	10.3892/or.2015.3982	53	5.30
TEY J (25), MEDICINE	10.1097/MD.0000000000000118	52	4.73
TEY J (6), ONCOTARGET	10.18632/oncotarget.15554	52	6.50
JU CY (26), SMALL	10.1002/smll.201804191	50	8.33
ZHANG FF (27), J EXP CLIN CANC RES	10.1186/s13046-018-0821-4	46	6.57

The **Table 8** also lists the top 20 most cited articles in the field of gastric cancer radiotherapy (6, 10, 11, 14, 16, 25, 28–41).These articles better reflect the research advancements in radiotherapy for gastric cancer treatment.

**Table 8:** Summarizes the top 20 most cited articles in the field of gastric cancer radiotherapy.

Document	DOI	Citations
PARK SH (11), J CLIN ONCOL	10.1200/JCO.2014.58.3930	74
CATS A (10), LANCET ONCOL	10.1016/S1470-2045(18)30132-3	53
LEONG T (14), BMC CANCER	10.1186/s12885-015-1529-x	26
TEY J (25), MEDICINE	10.1097/MD.0000000000000118	24
YU IJ (28), RADIOTHER ONCOL	10.1016/j.radonc.2015.08.009	16
KONDOH C (29), BMC PALLIAT CARE	10.1186/s12904-015-0034-y	14
FUCHS CS (16), J CLIN ONCOL	10.1200/JCO.2017.74.2130	14
TEY J (6), ONCOTARGET	10.18632/oncotarget.15554	14
DAI Q (30), J SURG ONCOL	10.1002/jso.23795	12
TRIP AK (31), RADIOTHER ONCOL-a	10.1016/j.radonc.2014.05.003	11
STUMPF PK (32), CANCER-AM CANCER SOC	10.1002/cncr.30748	10
LEE YH (33), BMC CANCER	10.1186/s12885-017-3508-x	10
TEY J (34), CANCER MED-US	10.1002/cam4.2021	10
TRIP AK (31), RADIOTHER ONCOL	10.1016/j.radonc.2014.08.039	9
HIRAMOTO S (35), INT J CLIN ONCOL	10.1007/s10147-018-1317-0	9
STIEKEMA J (36), ANN SURG ONCOL	10.1245/s10434-013-3397-4	8
KIM MS (37), WORLD J GASTROENTERO	10.3748/wjg.v21.i9.2711	8
KAWABATA H (38), J PALLIAT MED	10.1089/jpm.2016.0141	8
WANG X (39), BRIT J CANCER	10.1038/bjc.2017.424	8
LIU GFF (40), PLOS ONE	10.1371/journal.pone.0082642	7

### 3.5 Keywords

**Figure 8:**
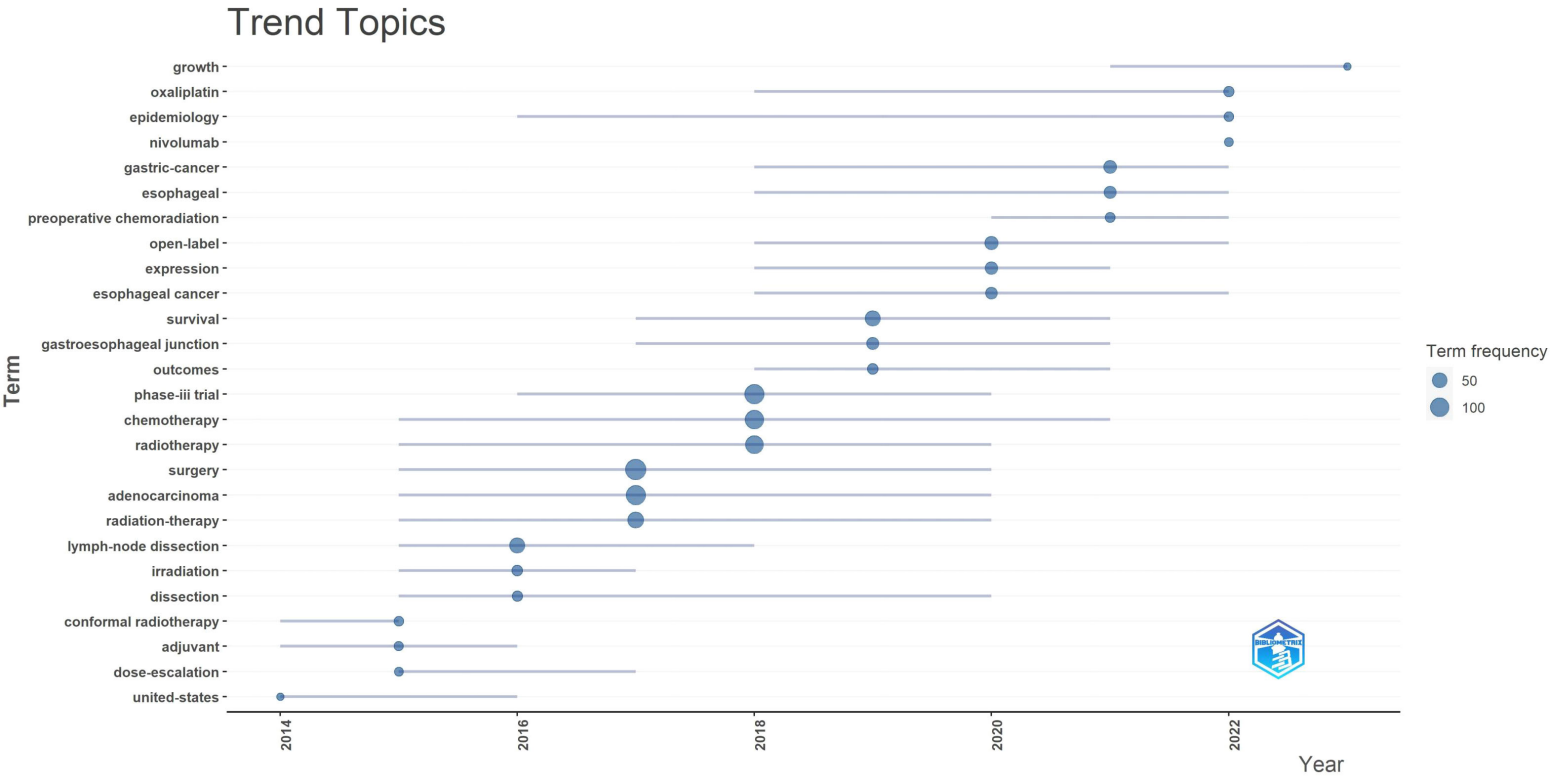
Trend topics over time.

Keywords are crystallizations of the content of an article, possessing high generality and reflective power within a particular research field, directly pointing to the essence of the text. Therefore, commonly used high-frequency keywords can present the focal issues of a research field, macroscopically reflecting the research hotspots within a certain period. To depict the current research status and hotspots in the field of gastric cancer radiotherapy, frequencies of keywords and the emergence of hot topics in different time periods were analyzed using R Studio (**Table 9**, **Figure 8**).

**Table 9:** Summarizes the top 50 most frequent words in the field of gastric cancer radiotherapy.

Rank	Words	Occurrences	Rank	Words	Occurrences
1	surgery	140	26	neoadjuvant chemotherapy	29
2	adenocarcinoma	119	27	expression	28
3	phase-iii trial	118	28	esophageal	26
4	chemotherapy	100	29	radiation	26
5	radiotherapy	91	30	gastroesophageal junction	25
6	chemoradiotherapy	86	31	intensity-modulated radiotherapy	24
7	perioperative chemotherapy	66	32	d2 gastrectomy	22
8	radiation-therapy	61	33	esophageal cancer	22
9	therapy	58	34	patterns	21
10	carcinoma	56	35	randomized-trial	21
11	lymph-node dissection	53	36	management	19
12	survival	53	37	recurrence	17
13	chemoradiation	43	38	metaanalysis	16
14	stomach	42	39	squamous-cell carcinoma	16
15	capecitabine	39	40	apoptosis	15
16	gastrectomy	38	41	irradiation	15
17	trial	38	42	outcomes	15
18	resection	37	43	dissection	14
19	cancer	36	44	double-blind	14
20	cisplatin	36	45	oxaliplatin	14
21	preoperative chemoradiotherapy	35	46	phase-ii	14
22	open-label	33	47	radiochemotherapy	14
23	adjuvant chemotherapy	30	48	1st-line therapy	13
24	gastric-cancer	30	49	impact	13
25	curative resection	29	50	s-1	13

After searching for the terms “gastric cancer” and “radiotherapy,” analysis of the top 50 high-frequency keywords reveals that “surgery,” “adenocarcinoma,” “phase-III trial,” and “chemotherapy” are the most frequently appearing terms, with 140, 119, 118, and 100 occurrences respectively. This indicates that adenocarcinoma is the predominant pathological type of gastric cancer, with surgery and chemotherapy currently being the mainstream treatments. Radiotherapy continues to play a supportive role in gastric cancer treatment, as complete pathological remission is challenging to achieve solely with radiotherapy. Phase-III clinical trials remain the most evidence-based approach among all articles. The top 25 keywords mainly focus on gastric cancer treatments involving radiotherapy, chemotherapy, and surgery. Specific hotspots related to gastric cancer radiotherapy are reflected in keywords ranked 26-50, such as “neoadjuvant radiochemotherapy,” “gastroesophageal,” and “intensity-modulated radiotherapy.”

**Figure 9:**
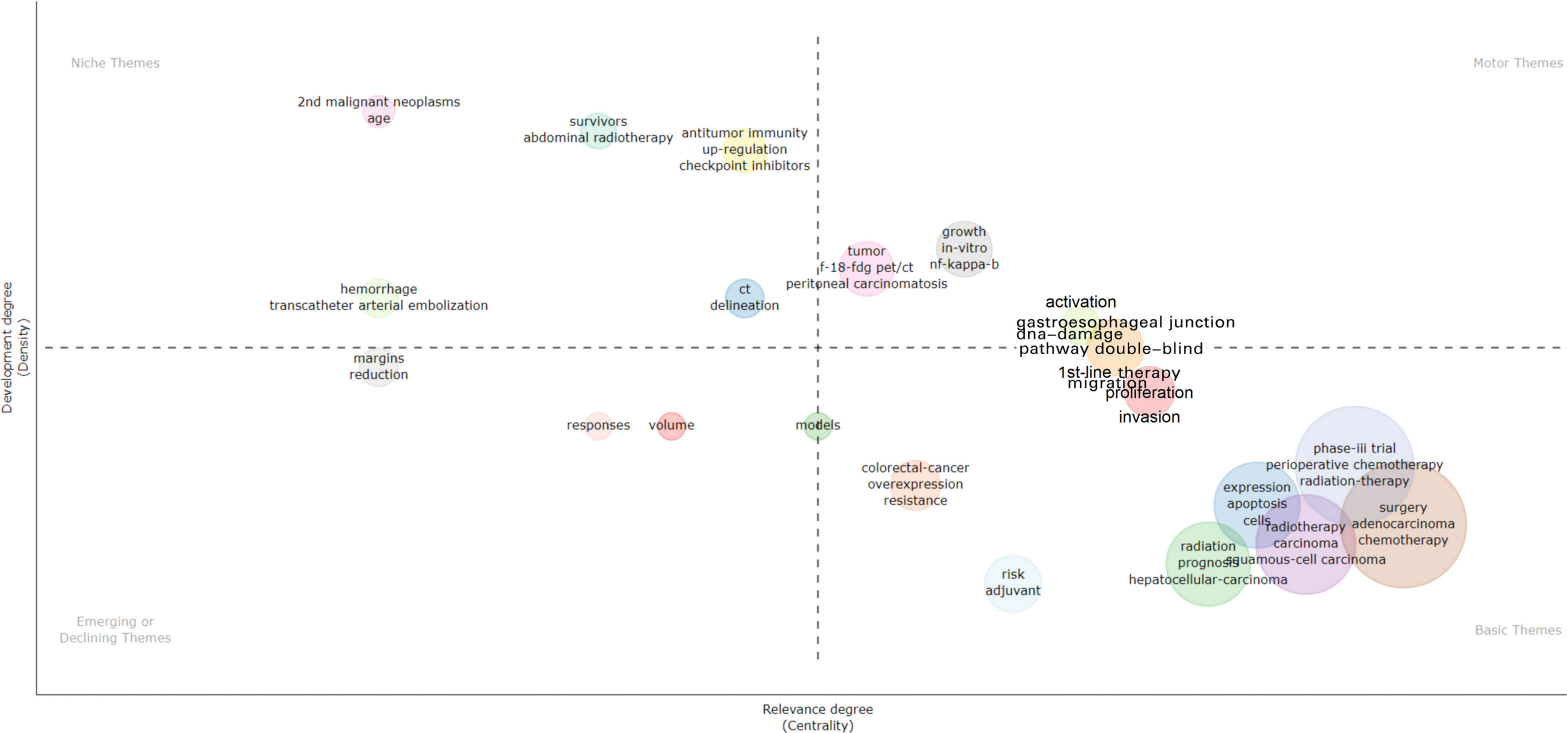
Thematic map.

The main themes and trends are depicted in the **Figure 9**. In the thematic map, the x-axis represents centrality, which indicates the importance of the field, while the y-axis represents density, which indicates the potential for development. Based on this, four quadrants can be drawn:•First quadrant (top-right corner): motor-themes, which are both important and well-developed. Second quadrant (top-left corner): very specialized/niche themes, which are well-developed but not important for the current field. Third quadrant (bottom-left corner): emerging or disappearing themes, which are marginal and underdeveloped, possibly newly emerging or about to disappear. Fourth quadrant (bottom-right corner): basic themes, which are important for the field but have not yet been well-developed. These generally refer to fundamental concepts.

The thematic map indicates emerging topics such as radiotherapy dose and treatment response, while clinical trials (survival, phase III trials) and treatment strategies (surgery, chemotherapy, radiotherapy, perioperative treatment) represent fundamental and cross-sectional themes. Checkpoint inhibitors and bleeding are isolated topics.

### 3.6 Collaboration

**Figure 10:**
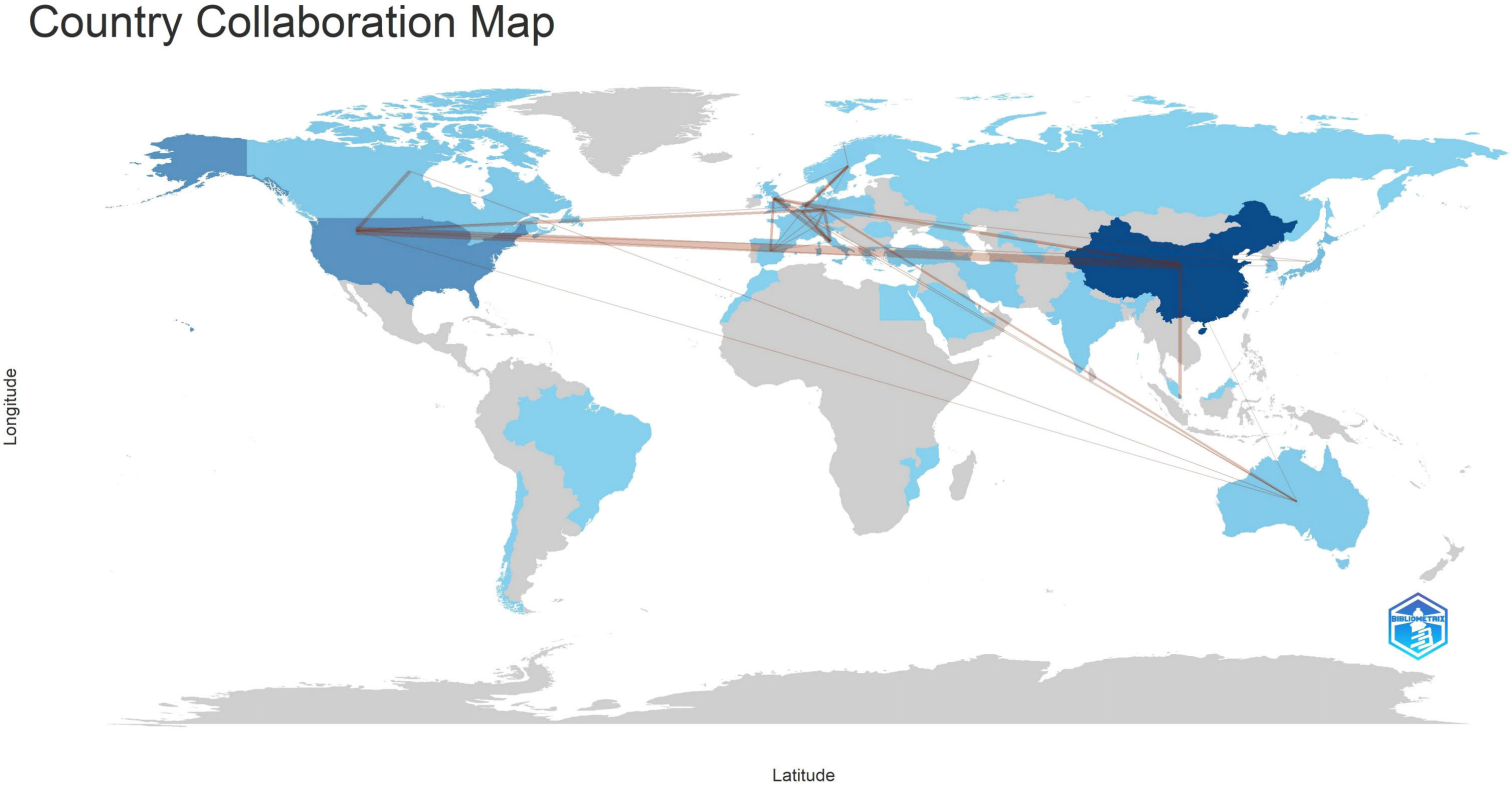
Countries’ collaboration world map.

The national collaboration network (**Figure 10**) shows country-based collaborations based on publications. The thickness of lines represents the proximity of collaboration. Close collaborations are observed between the United States and Canada, United States and China, China and Singapore, as well as Germany and Australia. There is a clear tendency for collaboration among European countries.

## 4 Discussion

In our bibliometric analysis of scientific literature on gastric cancer radiotherapy from 2014 to 2023, we observed an average annual growth rate of approximately -2.05%, indicating challenges in radiotherapy for gastric cancer treatment. The anatomical characteristics of the stomach, partially obscured by the liver and its frequent peristalsis, all limit the efficacy of radiotherapy. Additionally, since gastric cancer is predominantly adenocarcinoma, its sensitivity to radiotherapy is lower compared to squamous cell carcinoma. So, there were generally fewer articles discussing gastric cancer radiotherapy. With the results of several large-scale clinical trials on gastric cancer radiotherapy (such as the CRITICS and the ARTIST) over the past decade yielding less-than-satisfactory outcomes, the research interest in gastric cancer radiotherapy has declined.

### 4.1 Countries and institutions

Analyzing national and institutional distributions allows us to identify major contributors in this field. China leads in publication quantity, benefiting from East Asia’s highest gastric cancer incidence and its large population of patients. Moreover, China’s numerous doctors and several top institutions involved in gastric cancer radiotherapy research contribute significantly to its high productivity. However, the institution with the most publications is Samsung Medical Center in South Korea, highlighting Korean institutions’ deep exploration in this medical domain. Two of the top five highly cited articles in gastric cancer radiotherapy originate from South Korea, demonstrating its significant influence in this field. Despite China’s relatively late start in this field compared to South Korea, its development pace has been remarkable, with both quantity and quality of publications steadily increasing in recent years. These achievements stem from comprehensive advancements in Chinese healthcare, although their impact still requires further enhancement.

The geographical distribution of scientific publications spans continents, reflecting gastric cancer’s high incidence globally. Primary scientific production centers are in Asia (China, South Korea), North America (United States, Canada), and Europe (Netherlands, Germany, Italy). South Korea and China exhibit high quantitative scientific output, showcasing concentrated rather than dispersed scientific capabilities. North American and European countries maintain high scientific productivity, often engaging in regional scientific collaborations. The geographic distribution of scientific outputs highlights top academic institutions in gastric cancer radiotherapy across different regions. One limitation of the current analysis is that collaboration is measured simplistically using shared authors, which may not fully capture active scientific networks or the scientific value of published works.

### 4.2 Journals

By analyzing journal sources, researchers can efficiently identify suitable outlets for their papers. Journals like “Frontiers in Oncology” and “Cancer” have shown the highest output in recent years, indicating significant influence in gastric cancer radiotherapy and suitability for submission. The impact factors of these journals partly reflect the importance and priority of radiotherapy. Such high-quality journals underscore the significance of radiotherapy in gastric cancer as a vital research direction.

### 4.3 Authors

Authors frequently cited are considered more influential than less cited ones. In terms of contributions and citations, the most influential authors in this field are Jeeyun Lee from South Korea and Annomieke Cats from the Netherlands, both leading large-scale Phase III clinical trials in adjuvant radiotherapy and chemoradiotherapy for gastric cancer. Their teams represent excellent potential collaborators for researchers in gastric cancer radiotherapy studies.

### 4.4 Keywords

High-frequency keywords are pivotal in presenting hot topics within a research field, reflecting its evolving themes over different periods. From 2014 to 2016, gastric cancer radiotherapy primarily focused on “adjuvant therapy,” transitioning to “neoadjuvant chemoradiotherapy” from 2020 to 2022. Initially, studies emphasized radiotherapy forms (e.g., conformal radiotherapy) and dosages. Subsequently, due to the fixed location and high sensitivity of esophagogastric junction cancer, extensive research occurred from 2017 to 2019. In 2022, the advent of “nivolumab” marked the era of immunotherapy, resulting in a surge of articles on this topic. These developments illustrate discussions on treatment modalities, radiation patterns, treatment sites, and emerging combined therapies in gastric cancer radiotherapy.

### 4.5 Analysis of f highly cited articles

#### 4.5.1 Postoperative adjuvant therapies

Since 2014, research on radiotherapy for gastric cancer has evolved, starting with postoperative adjuvant therapies, prominently represented by the Korean ARTIST study (11). This large-scale prospective, randomized, multicenter phase III clinical trial led by Se Hoon Park et al. explored whether adding radiotherapy to adjuvant chemotherapy could enhance disease-free survival (DFS) for D2-resected gastric cancer (GC) patients. The results indicated significant DFS improvement in lymph node-positive and intestinal-type GC patients with combined radiochemotherapy. Similar trends were observed for DFS and overall survival (OS) stratified by disease stage. Subsequently, the ARTIST 2 trial was initiated to evaluate adjuvant chemotherapy and radiotherapy specifically for lymph node-positive, D2-resected GC patients.

The CRITICS study (10), An international, multicenter, open-label, randomized controlled, phase III clinical trial led by the Netherlands in 2018, employed a research design similar to that of the ARTIST study. It found that compared to postoperative chemotherapy alone, postoperative radiochemotherapy did not improve overall survival for resectable gastric cancer patients who had undergone adequate preoperative chemotherapy and surgery. Given poor patient compliance in both treatment groups, future research should focus on optimizing preoperative treatment strategies. Postoperative adjuvant radiotherapy for gastric cancer had once again encountered challenges. Some scholars argued that the role of radiotherapy was not fully realized in this study. The controversy surrounding the delineation of the radiation target area, along with a treatment completion rate of less than 50% among enrolled patients, may have undermined the survival benefits of postoperative radiochemotherapy. This study is an open-label trial, meaning both the patients and the researchers were aware of the treatment regimen administered. This design may introduce potential biases, representing another limitation of the study, which could affect the accuracy and generalizability of the results.

Subsequently, the results of the ARTIST 2 study (42) published in 2020 indicated that for patients with stage II and III gastric cancer with positive lymph nodes, surgery plus SOX or SOXRT prolonged disease-free survival (DFS) compared to surgery plus S1. However, there was no significant reduction in local recurrence when comparing postoperative SOXRT with the postoperative SOX regimen, with both exhibiting similar DFS outcomes. This suggests that for stage II and III gastric cancer following D2 radical surgery, the SOX regimen or other combinations of fluoropyrimidine and oxaliplatin may serve as standard treatment. Postoperative chemoradiotherapy has still not been included as a standard treatment.

#### 4.5.2 Neoadjuvant therapies

Due to suboptimal outcomes with postoperative radiotherapy, investigations have shifted towards preoperative radiochemotherapy models, exemplified by studies such as RTOG 9904 (43), CROSS (44), POET (45), and TOP_GEAR (14), aiming to provide high-level evidence for preoperative radiotherapy in gastric cancer treatment. RTOG 9904, an early-phase study, included 49 localized gastric cancer patients who received induction chemotherapy followed by synchronous fluoropyrimidine-based radiotherapy (45 Gy in 25 fractions). It reported pathologic complete response (pCR) and R0 resection rates of 26% and 77%, respectively. The CROSS study compared surgery alone with neoadjuvant radiochemotherapy plus surgery in 366 patients with esophageal or esophagogastric junction tumors. Results showed improved survival outcomes in the radiochemotherapy group (48.6 months vs. 24.0 months, P=0.003), with a higher R0 resection rate (92% vs. 69%). Long-term results from this study consistently suggested that radiochemotherapy provides better survival benefits. However, the study’s limitation lies in significant heterogeneity among enrolled patients, including various histological types such as adenocarcinoma, squamous cell carcinoma, and unspecified types, influencing treatment response and prognosis.

The POET study included 119 locally advanced esophagogastric junction tumor patients, comparing the efficacy of preoperative chemotherapy versus preoperative radiochemotherapy. Results showed significantly higher pCR rates (15.6% vs. 2.0%, P=0.03) and tumor-free lymph node rates (64.4% vs. 36.7%, P=0.001) in the radiochemotherapy group. Long-term outcomes demonstrated superior 3-year and 5-year survival rates in the radiochemotherapy group. Current research primarily focuses on esophagogastric junction tumors regarding preoperative radiotherapy, which notably enhances pCR and R0 resection rates. However, whether the increased pCR in initially resectable patients translates into survival benefits requires further confirmation.

#### 4.5.3 Palliative therapies

Palliative radiotherapy for gastric cancer remains a research hotspot. Jeremy Tey et al. (6, 25, 34) highlighted the efficacy and good tolerability of 3D conformal external beam radiotherapy for local palliative treatment, which can extend patient survival. Short-term (39 Gy BED) radiotherapy plans are effective in symptom relief for these patients.

Overall, advancements in radiotherapy for locally advanced gastric cancer benefit potentially resectable, unresectable, and isolated distant metastatic patients alike. With the advent of immunotherapy, combined radiotherapy and immunotherapy may usher in new breakthroughs, as radiotherapy could enhance tumor cell sensitivity to immunotherapy. Some studies have reported promising pCR rates as high as 38.2% (46), indicating significant potential for immunotherapy-radiotherapy combinations.

## 5 Conclusion

The evolving objectives of research in radiotherapy reflect the advancements in global gastric cancer oncology over time. Current management of gastric cancer is based on multidisciplinary integration of treatments, including surgery, radiotherapy, chemotherapy, targeted therapy, and immunotherapy. Radiotherapy offers benefits to gastric cancer patients with potentially resectable, unresectable, or isolated distant metastases. The combination of immunotherapy with radiotherapy holds significant potential and may represent a new breakthrough in treatment.

## Data Availability

The raw data supporting the conclusions of this article will be made available by the authors, without undue reservation.
